# Oxidative stress‐related cellular aging causes dysfunction of the Kv3.1/KCNC1 channel reverted by melatonin

**DOI:** 10.1111/acel.14185

**Published:** 2024-05-09

**Authors:** Sara Spinelli, Alessia Remigante, Raffaella Liuni, Gianluca Mantegna, Giuseppe Legname, Angela Marino, Rossana Morabito, Silvia Dossena

**Affiliations:** ^1^ Department of Chemical, Biological, Pharmaceutical and Environmental Sciences University of Messina Messina Italy; ^2^ Institute of Pharmacology and Toxicology Paracelsus Medical University Salzburg Austria; ^3^ Laboratory of Prion Biology, Department of Neuroscience Scuola Internazionale Superiore di Studi Avanzati (SISSA) Trieste Italy; ^4^ Research and Innovation Center Regenerative Medicine and Novel Therapies (FIZ RM and NT) Paracelsus Medical University Salzburg Austria

**Keywords:** cellular aging, d‐galactose, Kv3.1/KCNC1, melatonin, oxidative stress, voltage‐gated potassium channels

## Abstract

The voltage‐gated Kv3.1/KCNC1 channel is abundantly expressed in fast‐spiking principal neurons and GABAergic inhibitory interneurons throughout the ascending auditory pathway and in various brain regions. Inactivating mutations in the *KCNC1* gene lead to forms of epilepsy and a decline in the expression of the Kv3.1 channel is involved in age‐related hearing loss. As oxidative stress plays a fundamental role in the pathogenesis of epilepsy and age‐related hearing loss, we hypothesized that an oxidative insult might affect the function of this channel. To verify this hypothesis, the activity and expression of endogenous and ectopic Kv3.1 were measured in models of oxidative stress‐related aging represented by cell lines exposed to 100 mM d‐galactose. In these models, intracellular reactive oxygen species, thiobarbituric acid reactive substances, sulfhydryl groups of cellular proteins, and the activity of catalase and superoxide dismutase were dysregulated, while the current density of Kv3.1 was significantly reduced. Importantly, the antioxidant melatonin reverted all these effects. The reduction of function of Kv3.1 was not determined by direct oxidation of amino acid side chains of the protein channel or reduction of transcript or total protein levels but was linked to reduced trafficking to the cell surface associated with Src phosphorylation as well as metabolic and endoplasmic reticulum stress. The data presented here specify Kv3.1 as a novel target of oxidative stress and suggest that Kv3.1 dysfunction might contribute to age‐related hearing loss and increased prevalence of epilepsy during aging. The pharmacological use of the antioxidant melatonin can be protective in this setting.

## Graphical abstract

The voltage‐gated Kv3.1/KCNC1 channel is expressed in fast‐spiking GABAergic inhibitory interneurons and principal neurons of the ascending auditory pathway and dysfunction of this channel leads to epilepsy and age‐related hearing loss. Here we show that the ion current of endogenous and ectopically expressed Kv3.1 is significantly inhibited in models of oxidative stress‐related cellular aging. Inhibition of Kv3.1 involved impaired trafficking to the plasma membrane associated with Src phosphorylation and endoplasmic reticulum stress and was prevented by pharmacological concentrations of melatonin.

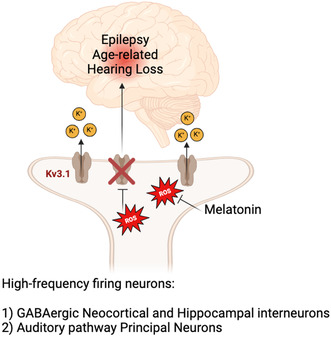

**Abbreviations**
CAT: catalase
Cav1: caveolin 1
CK2: casein kinase 2
DEE: developmental and epileptic encephalopathy
D‐Gal: d‐galactose
D‐Man: d‐mannitol
H_2_O_2_
: hydrogen peroxide
HEK: human embryonic kidney
iNOS: inducible nitric oxide synthase
Kv: voltage‐gated K
MDA: malondialdehyde
MNTB: medial nucleus of the trapezoid body
NOX: NADPH oxidase
P2A: 2A peptide
PBS: phosphate‐buffered solution
PKC: protein kinase C
ROS: reactive oxygen species
RT‐qPCR: real‐time PCR
SH: sulfhydryl
SOD: superoxide dismutase
TBARS: thiobarbituric acid reactive substances
TEA: tetraethylammonium
TNOS: total nitric oxide synthase

## 1 INTRODUCTION

Voltage‐gated K^+^ channels (Kv) represent a broad family of K^+^‐selective channels sensitive to the difference of potential across the cell membrane and pharmacologically distinct from other K^+^ channels in that they are blocked by micromolar concentrations of 4‐aminopyridine and millimolar or sub‐millimolar concentrations of tetraethylammonium (TEA). These channels are abundantly expressed in excitable cells and, being activated at depolarized membrane potentials, play a fundamental role in action potential repolarization. The alpha subunits of Kv channels form the ion permeation pathway and can assemble into homo‐ and heterotetrameric voltage‐gated potassium channels of which the activity is modulated by auxiliary beta subunits. Each alpha subunit comprises six transmembrane segments (S1–S6), with the S5–S6 segments forming the ion‐conducting pore domain and the S1–S4 segments forming the voltage‐sensing domain. The alpha subunits belong to 12 subfamilies (Kv1‐12), of which the members are grouped according to their electrophysiological properties and function into delayed rectifiers, A‐type, outward‐rectifying, inwardly‐rectifying, slowly activating, and modifiers/silencers (Gutman et al., 2005). Among these, the delayed rectifiers are characterized by a positively shifted voltage‐dependent activation, and their current is rapidly activating and slowly inactivating or non‐inactivating over time. Within the delayed rectifiers group, the Shaw‐related channels include the alpha subunit of Kv3.1, encoded by the *KCNC1* gene, mapped on human chromosome 11 (Grissmer et al., 1992).

Following alternative splicing, *KCNC1* gives rise to two transcripts that encode two protein isoforms, Kv3.1a and b respectively, which differ in their C‐terminus, with isoform a being shorter. In the avian auditory pathways, Kv3.1a expression appears very early during embryonic development, whereas Kv3.1b appears at later embryonal stages (Parameshwaran‐Iyer et al., 2003). In the rat brain, the two isoforms coexist and are co‐expressed in the same neurons, but Kv3.1a predominates during embryonal and early postnatal stages, while Kv3.1b surges between postnatal Days 7 and 14 and appears to be the predominant isoform in the adult life (Perney et al., 1992). The two variants exhibit indistinguishable electrophysiological properties, but Kv3.1b is regulated by protein kinase C (PKC)‐dependent phosphorylation of Ser503 on the C‐terminus, which decreases the current amplitude in the absence of stimuli in auditory neurons of the brainstem (Macica et al., 2003).

Kv3.1/KCNC1 is prominently expressed in high‐frequency firing neurons in various brain regions (cerebellum > globus pallidus, subthalamic nucleus, substantia nigra > reticular thalamic nuclei, cortical, and hippocampal interneurons > inferior colliculi, cochlear and vestibular nuclei) (Gutman et al., 2005). The voltage dependence of activation shifted toward potentials more positive than −20 mV and the fast deactivation rate upon repolarization enable the Kv3.1 channels to specifically activate during the action potential and accelerate action potential repolarization while minimizing the duration of the refractory period between subsequent action potentials. These peculiar biophysical properties are essential to permit high‐frequency repetitive firing of GABAergic neocortical and hippocampal interneurons and auditory nuclei principal neurons (Rudy & McBain, 2001) and are believed to be dependent on constitutive and abundant casein kinase 2 (CK2)‐mediated phosphorylation (Macica & Kaczmarek, 2001).

Consistent with its expression on GABAergic inhibitory interneurons, loss of function of Kv3.1 due to genetic mutation leads to various forms of myoclonic epilepsy (Cameron et al., 2019), pointing to the fundamental role of this channel in suppressing neuronal hyperexcitability. The expression of Kv3.1 on rapidly firing principal neurons of the ascending auditory pathway permits their rapid repolarization, which is essential for the generation of high‐frequency action potentials following stimulation at high sound frequencies (Peixoto Pinheiro et al., 2021). Importantly, the expression of Kv3.1 within the auditory pathway declines with age (Jung et al., 2005; Zettel et al., 2007), possibly contributing to age‐related hearing loss in humans (Peixoto Pinheiro et al., 2021).

Both epilepsy and hearing loss are oxidative stress‐related diseases and their prevalence increases with age. Although Alzheimer's disease and stroke contribute to the increased risk of seizures in the elderly, 30%–50% of cases of seizures in patients over 60 years of age are of unknown etiology (Ramsay et al., 2004; Tanaka et al., 2013). Oxidative stress is regarded as an important contributor to the pathogenesis of epilepsy and epileptogenesis, especially during aging. Increased levels of reactive oxygen species (ROS) can be generated in the brain after a seizure, and an elevated oxidative status can trigger subsequent seizures, thus generating a vicious circle (Patel, 2004; Puttachary et al., 2015). Chemoconvulsants utilized to obtain animal models of epilepsy generate intense ROS production and extensive oxidative or nitrative damage (Borowicz‐Reutt & Czuczwar, 2020). Accordingly, several studies found altered oxidative stress markers in the blood and red blood cells of epileptic patients (Ethemoglu et al., 2021; Wang et al., 2021).

Oxidative stress also plays a fundamental role in age‐related hearing loss. Mitochondrial dysfunction associated with excess ROS production and decreased antioxidant function play a central role in the aging process of cochlear cells including the hair cells of the organ of Corti and cells of the stria vascularis, afferent spiral ganglion neurons, and the central auditory pathways (Fujimoto & Yamasoba, 2014; Someya & Prolla, 2010). Accordingly, reduction of oxidative stress prevented age‐related hearing loss in mice (Someya et al., 2010). In this context, specific K^+^ channels, and particularly Kv3.1, have been suggested as early targets susceptible to oxidative damage (Peixoto Pinheiro et al., 2021).

d‐galactose (D‐Gal) has been extensively utilized to obtain cell‐based and animal models of oxidative stress‐related aging that are considered a good representation of natural aging (Azman & Zakaria, 2019; Ji et al., 2017). Growing evidence indicates that D‐Gal induces oxidative stress following events occurring in the intracellular environment. Indeed, D‐Gal can cross plasma membranes via glucose transporters. Physiological concentrations of D‐Gal are metabolized to galactitol and galactose‐1‐phosphate by aldose reductase and galactokinase, respectively. Galactitol is excreted into the urine, while galactose‐1‐phosphate can be converted to glucose‐6‐phosphate and flow into glycolysis (Conte et al., 2021). In the presence of excess galactose, alternative pathways that usually play marginal roles in cellular metabolism can be substantially activated. D‐Gal can be converted to galactonate, which is further metabolized to enter the pentose phosphate pathway. In addition, excess D‐Gal can be oxidized by galactose oxidase to produce hydrogen peroxide (H_2_O_2_), which can react with ferrous iron to generate hydroxyl radicals. Both H_2_O_2_ and hydroxyl radicals cause oxidative stress and direct damage to cellular components. Oxidative stress can also result from mitochondrial dysfunction as a consequence of D‐Gal‐induced inhibition of respiratory chain enzymes as well as from the accumulation of galactitol. Overall, D‐Gal‐induced oxidative stress was documented by elevation of oxidative stress markers including lipid peroxidation, malondialdehyde (MDA), nitrite, H_2_O_2_, 8‐oxoguanine, NADPH oxidase (NOX), total nitric oxide synthase (TNOS), inducible nitric oxide synthase (iNOS), ROS, and protein carbonylation in various animal and cellular models. In parallel, D‐Gal also led to a decrease in the activity of antioxidant compounds and enzymes, including glutathione, catalase (CAT), superoxide dismutase (SOD), glutathione‐S‐transferase, glutathione peroxidase, and total antioxidant capacity in these models (Azman & Zakaria, 2019). The D‐Gal mouse is a well‐established model of age‐related hearing loss. D‐Gal‐treated mice or rats develop premature hearing loss associated with oxidative stress, mitochondrial dysfunction, apoptosis within the stria vascularis, and cochlear synaptopathy (Du et al., 2020; Peng et al., 2023). Recently, we have set up a D‐Gal‐based, oxidative stress‐related model of aging in human erythrocytes, which is characterized by prominent oxidative stress on cellular proteins and lipids and functional dysregulation of the anion exchanger SLC4A1/AE1/band3, the main ion transport system in erythrocytes (Remigante et al., 2022). Here, we transposed this model to nucleated cells to explore whether Kv3.1 function can be affected by age‐related oxidative stress and test the pharmacological use of melatonin as a possible protective strategy.

Melatonin is the pleiotropic hormone produced by the pineal gland and plays a variety of receptor‐mediated and receptor‐independent physiological functions, including the regulation of the circadian rhythm and antioxidant activity, respectively. In various cell‐based and animal models of oxidative stress‐related diseases, melatonin directly neutralized free radicals and reactive oxygen and nitrogen species and stimulated the activity of SOD, glutathione peroxidase, and glutathione reductase (Reiter et al., 2000). In clinical trials, melatonin intake increased the total antioxidant capacity, SOD, glutathione, glutathione peroxidase, and glutathione reductase activities, and reduced MDA and protein carbonylation levels (Ghorbaninejad et al., 2020; Morvaridzadeh et al., 2020).

The findings presented here show for the first time that the ion current of endogenous and ectopically expressed Kv3.1 is significantly inhibited in cellular models of oxidative stress‐related aging prior to loss of cell viability. Inhibition of Kv3.1 occurred with no evidence of direct oxidation of the channel protein and most likely involved impaired trafficking to the plasma membrane. Pharmacological concentrations of melatonin prevented Kv3.1 dysfunction.

## 2 MATERIALS AND METHODS

### 2.1 Cell culture and treatment

Human embryonic kidney (HEK) 293 Phoenix cells, which are derived from 293 T cells, were cultured in Minimum Essential Eagle Medium (M5650, Sigma‐Aldrich) supplemented with 10% fetal bovine serum, 2 mM L‐glutamine, 100 U/mL penicillin, 100 μg/mL streptomycin and 1 mM pyruvic acid (sodium salt). NIH/3 T3 cells (mouse fibroblasts, CRL‐1658, directly obtained from ATCC) were cultured in Dulbecco's modified Eagle's Medium (D7777, Sigma‐Aldrich) supplemented with 10% newborn bovine serum (Gibco™), 100 U/mL penicillin and 100 μg/mL streptomycin.

Cells were maintained at 37°C, 5% CO_2_, 95% air, and 100% humidity. Subcultures were routinely established every second to third day by seeding the cells into 100 mm diameter Petri dishes following trypsin/ethylenediaminetetraacetic acid (EDTA) treatment.

For experiments, 100 mM D‐Gal with or without 100 μM melatonin was added to the cell culture medium for 3–72 h. For controls, 100 mM d‐mannitol (D‐Man) was used to obtain equal osmolarity of the cell culture medium.

### 2.2 Cell transfection and plasmid vectors

For transfection, NIH/3 T3 cells were seeded into 35 mm diameter Petri dishes and grown overnight to ~30% confluence. Cells were transfected with 3 μg of plasmid DNA and 6 μL of Metafectene Pro (Biontex, Munich, Germany), following the manufacturer's instructions. HEK 293 Phoenix cells were transfected with 3 μg of plasmid DNA using the calcium‐phosphate co‐precipitation method.

The plasmid vector pcDNA3.1 + P2A‐eGFP encoding for human Kv3.1a (KCNC1, NCBI Sequence ID: NM_004976.4) was purchased from GeneScript (USA), amplified in‐house, and sequenced for verification (Microsynth AG). The self‐cleaving 2A peptide (P2A) allows for the simultaneous expression of Kv3.1 and eGFP proteins from a single bicistronic mRNA without the production of fusion proteins. Control cells were transfected with an empty vector encoding EGFP only. For patch‐clamp, single transfected cells could be identified optically by detecting the fluorescent signal emitted by eGFP (excitation maximum, 488 nm; emission maximum, 507 nm). Experiments were performed 24–72 h after transfection.

### 2.3 Cell viability assay

Cell viability was evaluated by the CellTiter 96® AQueous One Solution Cell Proliferation Assay system (Promega, Madison, WI, USA), according to the manufacturer's instructions. Details are given in the Supplemental Information—Data S1.

### 2.4 Determination of thiobarbituric acid reactive substances

Thiobarbituric acid reactive substances (TBARS) levels were evaluated as formerly described (Mendanha et al., 2012) with minor modifications. Details are given in the Supplemental Information—Data S1.

### 2.5 Determination of total sulfhydryl group content

Total sulfhydryl (‐SH) group content was evaluated as formerly described (Aksenov & Markesbery, 2001), with minor modifications. Details are given in the Supplemental Information—Data S1.

### 2.6 Determination of intracellular reactive oxygen species

Total intracellular ROS levels were evaluated with the 2′,7′‐dichlorofluorescein diacetate indicator (H2DCFDA, D6883, Sigma‐Aldrich). Details are given in the Supplemental Information—Data S1.

### 2.7 Catalase and superoxide dismutase activity assay

Catalase (CAT) and superoxide dismutase (SOD) activity was evaluated by commercial kits (Catalase Assay Kit, MAK381, and Superoxide Dismutase Activity Assay Kit, CS0009, Sigma‐Aldrich), according to the manufacturer's instructions. Details are given in the Supplemental Information—Data S1.

### 2.8 Patch‐clamp experiments

Cells for electrophysiology were seeded on glass coverslips (diameter, 10 mm) contained in 30 mm diameter Petri dishes and grown overnight. Single cells were selected by phase contrast or fluorescence microscopy as appropriate and voltage‐clamped using the nystatin‐perforated whole‐cell patch‐clamp technique with pipette‐filling and bath solutions appropriately designed to isolate K^+^ currents. Further details are given in the Supplemental Information—Data S1. For data acquisition, EPC‐10 amplifiers (HEKA, Stuttgart, Germany) controlled by Macintosh computers running the Patch Master software (HEKA) were used. Fast and slow capacitance currents were monitored and compensated throughout the recordings. Access resistance was monitored and kept <20 mΩ. All current measurements were filtered at 3 kHz and digitized at 50 kHz. For data analysis, Fit Master (HEKA) and Excel (Microsoft) software were used. The current values (pA) were normalized to the membrane capacitance (pF) to obtain the current density (pA/pF), which is a measure of the current magnitude independent of the cell size. All experiments were carried out at room temperature.

### 2.9 Reverse transcription and quantitative real‐time PCR

Total RNA from human whole kidney and small intestine were purchased from Ambion (Foster City, CA, USA) and Clontech (Mountain View, CA, USA), respectively, and reverse‐transcribed in‐house. The frontal cortex tissue samples were from subjects who died from a pathology not affecting the brain and lacking any neurological signs and were originally obtained from the MRC Edinburgh Brain Bank. The main characteristics of these subjects are given in Table S1. The total RNA was isolated and reverse‐transcribed following a previously described protocol (Zattoni et al., 2022). The use of these samples was covered by ethical approval from the East of Scotland Research Ethics Service REC 1 (reference number 16/ES/0084). Informed consent for the research use of autopsy tissues was obtained from the relatives. Extraction of total RNA from cultured cells was performed with the RNeasy micro kit (Qiagen, Hilden, Germany) from cells grown to confluence on one 10 mm diameter or two 3.5 mm diameter Petri dishes. One μg of total RNA was used for the reverse transcription reaction with the QuantiTect® reverse transcription kit for cDNA synthesis with integrated removal of genomic DNA contamination (Qiagen). PrimeTime® qPCR primers for detecting Kv3.1/KCNC1, Kv1.1/KCNA1, Kv1.2/KCNA2, Kv1.3/KCNA3, Kv1.4/KCNA4, Kv1.6/KCNA6, Kv3.3/KCNC3, Kv3.4/KCNC4, and Kv4.1/KCND1 transcripts, as well as the housekeeping transcript *POLR2A*, were purchased from Integrated DNA Technologies (Coralville, IA, USA). The primer sequences are given in the Supplemental Table S2. qPCR reactions contained 5 μL of 1:10 cDNA template in a 20 μL final volume of 1X primers and 1X GoTaq® qPCR Master Mix (Promega, Madison, WI, USA). Real‐time PCR reactions were performed in technical duplicates for each sample, along with a minus reverse transcriptase control and no template control on the Rotor‐Gene (Qiagen) instrument. Transcript levels were normalized for those of the housekeeping transcript and analyzed with the comparative *Ct* method.

### 2.10 Western blot

To obtain crude cell lysates, cells were seeded in each well of a six‐well plate, transfected and/or treated as needed for 48 h, washed with phosphate‐buffered solution (PBS), and scraped in 100 μL of ice‐cold RIPA buffer (Sigma Aldrich) containing 1X Halt Protease Inhibitor Cocktail (Thermo Fisher Scientific) on ice. For Western blot on phosphorylated proteins, 1X Phosphatase Inhibitor Cocktail 2 (Sigma‐Aldrich) was added to the lysis buffer. Cell lysates were centrifuged at 900×*g* at 4°C for 10 min. The supernatant was collected and stored at −20°C until use.

To obtain each sample of plasma membrane proteins, cells were seeded in two Petri dishes of 15 cm diameter, treated as needed for 48 h, and merged in an individual cell pellet. Plasma membrane proteins were obtained with the Plasma Membrane Protein Extraction Kit (Amsbio, Cambridge, MA, USA), following the manufacturer's instructions.

Western blot was performed according to standard procedures. Further details, including all antibodies, are given in the Supplemental Methods.

### 2.11 Salts and chemicals

Melatonin (CAS Number: 73‐31‐4), D‐(+)‐Galactose (CAS Number: 59‐23‐4), and d‐mannitol (CAS Number: 69‐65‐8) were from Sigma‐Aldrich. Melatonin stock solutions were prepared in DMSO and stored protected from light. All salts and chemicals were *per analysis* grade.

### 2.12 Statistical analysis

All data were expressed as arithmetic means ± S.E.M. For statistical analysis, GraphPad Prism software (version 9.5.1 for Mac OS X, GraphPad Software Inc., CA, USA) was used. Significant differences between data sets were tested by two‐tailed, unpaired Student's *t* test or two‐way ANOVA with Bonferroni's or Dunnet's ad hoc post‐tests, as appropriate. Statistically significant differences between data sets were assumed at *p* < 0.05; (*n*) corresponds to the number of independent measurements.

## 3 RESULTS

### 3.1 Kv3.1a/KCNC1 is the predominant Kv3.1 variant expressed in HEK 293 Phoenix cells

**FIGURE 1:**
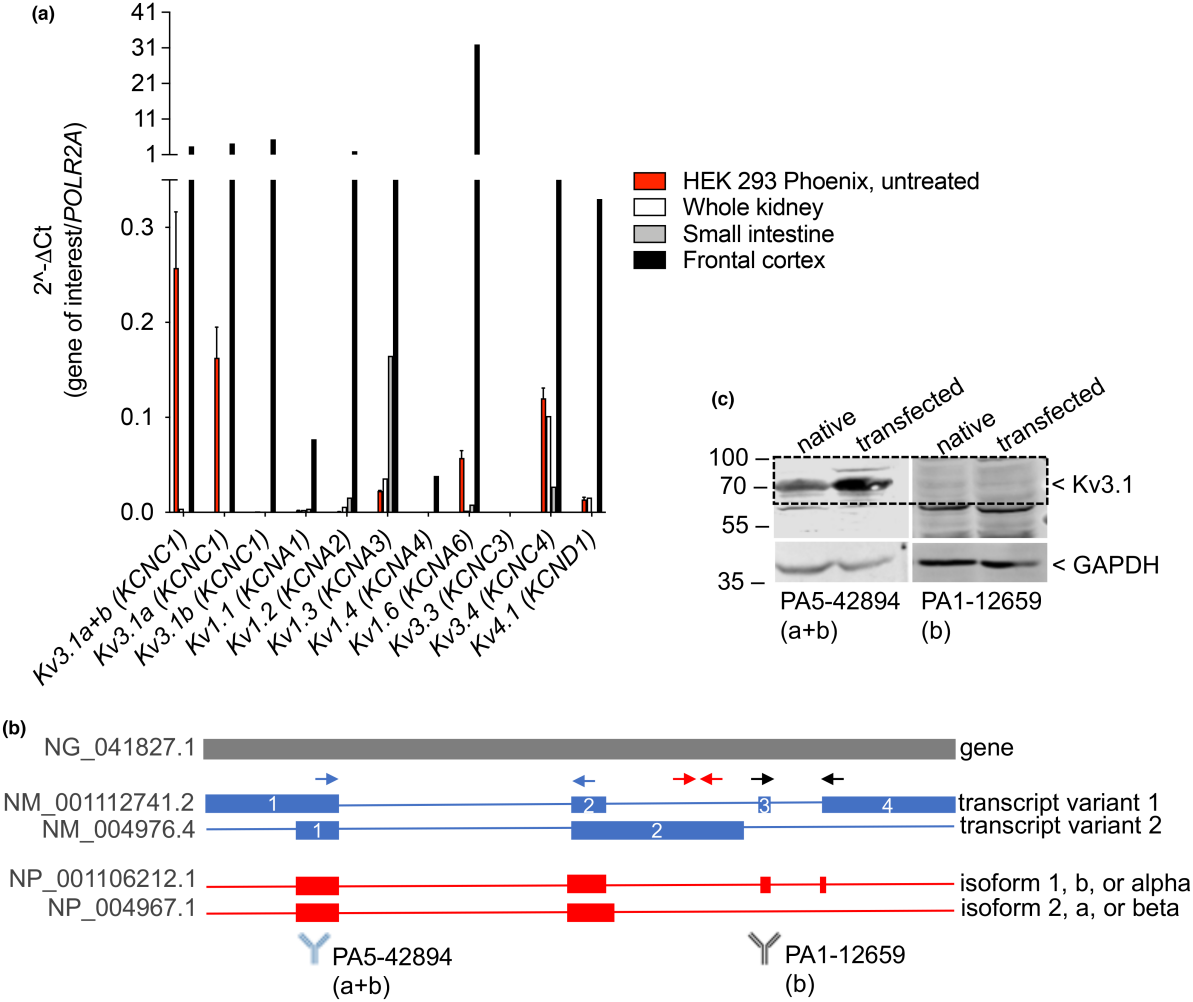
Expression profile of Kv channels in HEK 293 Phoenix cells. (a) Transcript levels of the indicated *Kv* (*KCN*) isoforms measured by qRT‐PCR in untreated HEK 293 Phoenix cells and normalized to those of the housekeeping transcript *POLR2A*. Human whole kidney, frontal cortex, and small intestine served as a control of primer efficiency or negative controls. Data are from three independent biological replicates, each run in technical duplicates. (b**)** Gene, transcript, and protein structure of Kv3.1/KCNC1. The NCBI genomic, transcript, and protein reference sequences are given. Primers spanning exons 1–2 and amplifying both transcript variants are indicated as blue arrows (primer set 1), primers annealing into exon 2 and specific for transcript variant 2 are indicated as red arrows (primer set 2), while primers spanning exons 3–4, and therefore specific for transcript variant 1, are indicated as black arrows (primer set 3). Similarly, the antibody against the N‐terminus of the protein and detecting both protein variants is represented in blue near the corresponding epitope, while the antibody directed against the C‐terminus, which is specific for isoform 1, is represented in black. (c) Total protein lysates from untransfected (native) cells or cells transfected with Kv3.1a were probed with the antibodies represented in panel b. The signal of the housekeeping protein GAPDH is also shown.

Screening of all nine *Kv/KCN* transcripts expected to be expressed in HEK 293 cells according to Zhang et al. (2022) by RT‐qPCR revealed that the most abundant Kv transcript in these cells corresponds to *Kv3.1/KCNC1*. Although less expressed, *Kv3.4/KCNC4* and *Kv1.6/KCNA6* were the second and third most abundant transcripts, respectively. Very low levels of *Kv1.3/KCNA3* and *Kv4.1/KCND1*, and virtually no expression of *Kv1.1/KCNA1*, *Kv1.2/KCNA2*, *Kv1.4/KCNA4*, and *Kv3.3/KCNC3* were detected (Figure 1a). The *Kv3.1/KCNC1* gene gives rise to two transcript variants, namely transcript variant 1 and transcript variant 2, encoding for a long protein variant, variably denoted in the literature as isoform 1, b, or alpha, and a shorter variant, where part of the Ct is missing, described as isoform 2, a, or beta (Figure 1b). To discriminate between these two transcripts, three Kv3.1 primer sets were utilized, that is primer set 1 (blue), spanning exons 1 and 2 and therefore recognizing both transcripts, primer set 2 (red), annealing into a region of exon 2 unique to transcript variant 2, and primer set 3 (black), spanning exons 3 and 4, and therefore recognizing only transcript variant 1 (Figure 1b). Primer set 3 gave no signal, denoting that the Kv3.1 signal obtained with primer set 1 corresponded to the sole transcript variant 2, which was also detected by primer set 2. Accordingly, an antibody directed against the Kv3.1 N‐terminus (amino acids 21–70) gave a clear band of the expected size in native HEK 293 Phoenix cells and cells transfected with a plasmid vector coding for Kv3.1a, while an antibody directed against the Kv3.1 C‐terminal portion (amino acids 567–585) gave no specific signal in these cells (Figure 1c). Therefore, Kv3.1a represents the main Kv3.1 transcript and protein variant in these cells.

Interestingly, we found the Kv3.1a transcript to be abundant in this sample of adult human frontal cortex (sample #19, Table S1), with an expression that was comparable to that of Kv3.1b (Figure 1a). This prompted us to investigate the expression of Kv3.1a in additional human brain samples (Table S1; Figure S1). We confirmed that Kv3.1a is significantly expressed (3–6 fold compared to the housekeeping gene *POLR2A*) in the adult human brain, although to a variable extent among subjects. However, Kv3.1b was confirmed as the predominantly expressed isoform, as reported in the literature (Perney et al., 1992).

### 3.2 Generation and characterization of an oxidative stress‐based model of cellular aging

**FIGURE 2:**
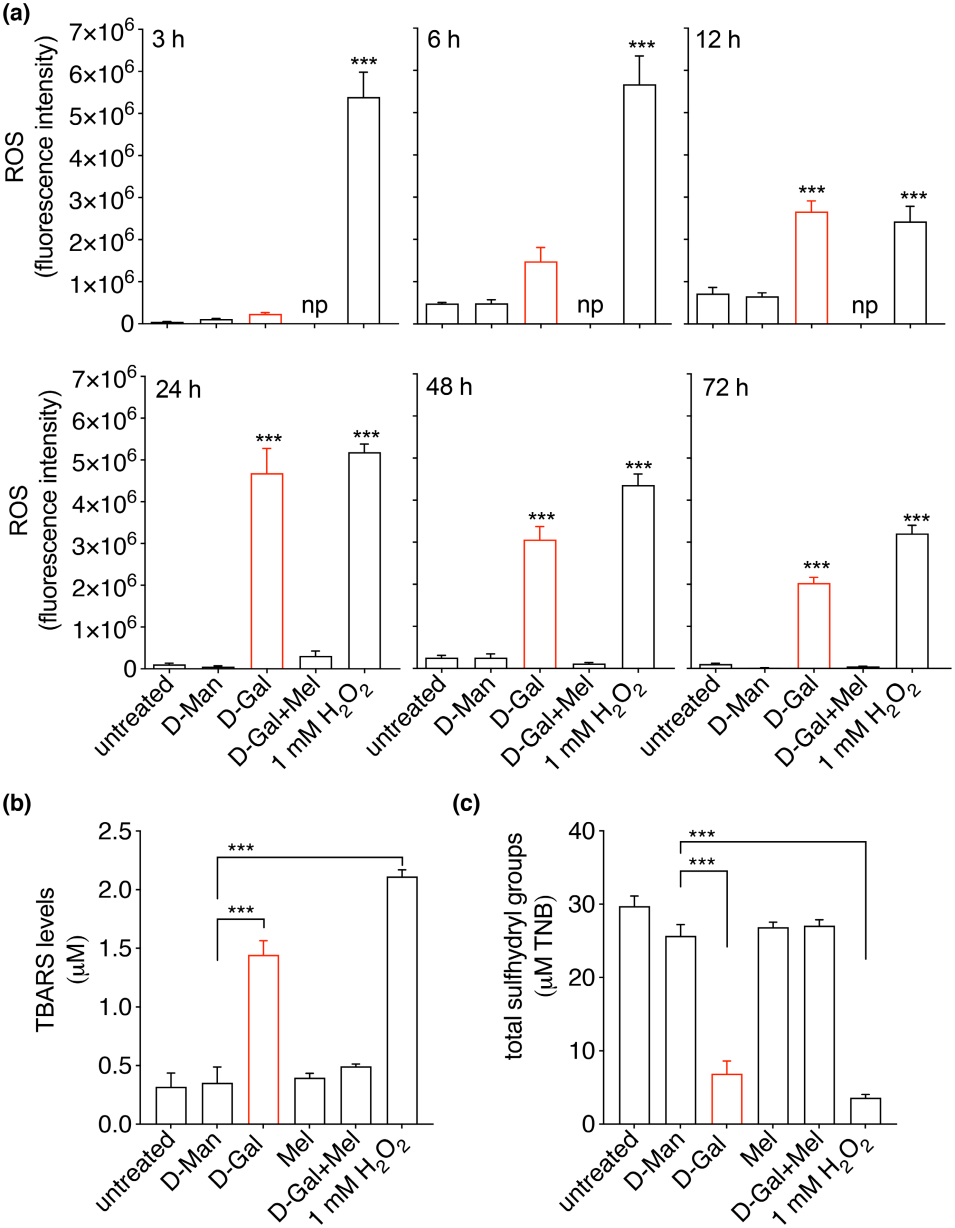
D‐Gal‐induced ROS and TBARS production, total protein sulfhydryl group oxidation, and protective effect of melatonin. (a) ROS levels (b**),** thiobarbituric acid reactive substances (TBARS), and (c) total protein sulfhydryl (SH) groups measured in untreated HEK 293 Phoenix cells or cells exposed to 100 mM D‐Man, D‐Gal, melatonin (Mel), or D‐Gal and 100 μM melatonin for 48 h or the indicated time. Cell treated with 1 mM H_2_O_2_ for 30 min were included as a positive control of ROS production and full oxidation of lipids and sulfhydryl groups, respectively. np, not performed, ***p < 0.001 versus D‐Man, one‐way ANOVA followed by Dunnett's post hoc test. Data are from 6 (for ROS) and 4 (for TBARS and SH groups) biological replicates.

To induce oxidative stress, HEK 293 Phoenix cells were left untreated or exposed to 100 mM D‐Gal in the presence or absence of 100 μM melatonin in the cell culture medium for 3–72 h. D‐Man, which is a cell‐impermeant, nonmetabolizable sugar, was utilized to obtain equal osmolarity of the cell culture medium in control samples. As expected, D‐Gal led to an elevation in the intracellular ROS content, which was statistically significant starting from 12 h of incubation. Importantly, pharmacological concentrations of melatonin in the presence of D‐Gal effectively prevented the ROS increase (Figure 2a).

In addition, exposure of cells to 100 mM D‐Gal for 48 h led to a significant elevation of TBARS content as well as a reduction in the total sulfhydryl (SH) groups, which denoted lipid peroxidation and oxidation of total cellular proteins, respectively. Again, these effects were prevented by melatonin (Figure 2b,c).

**FIGURE 3:**
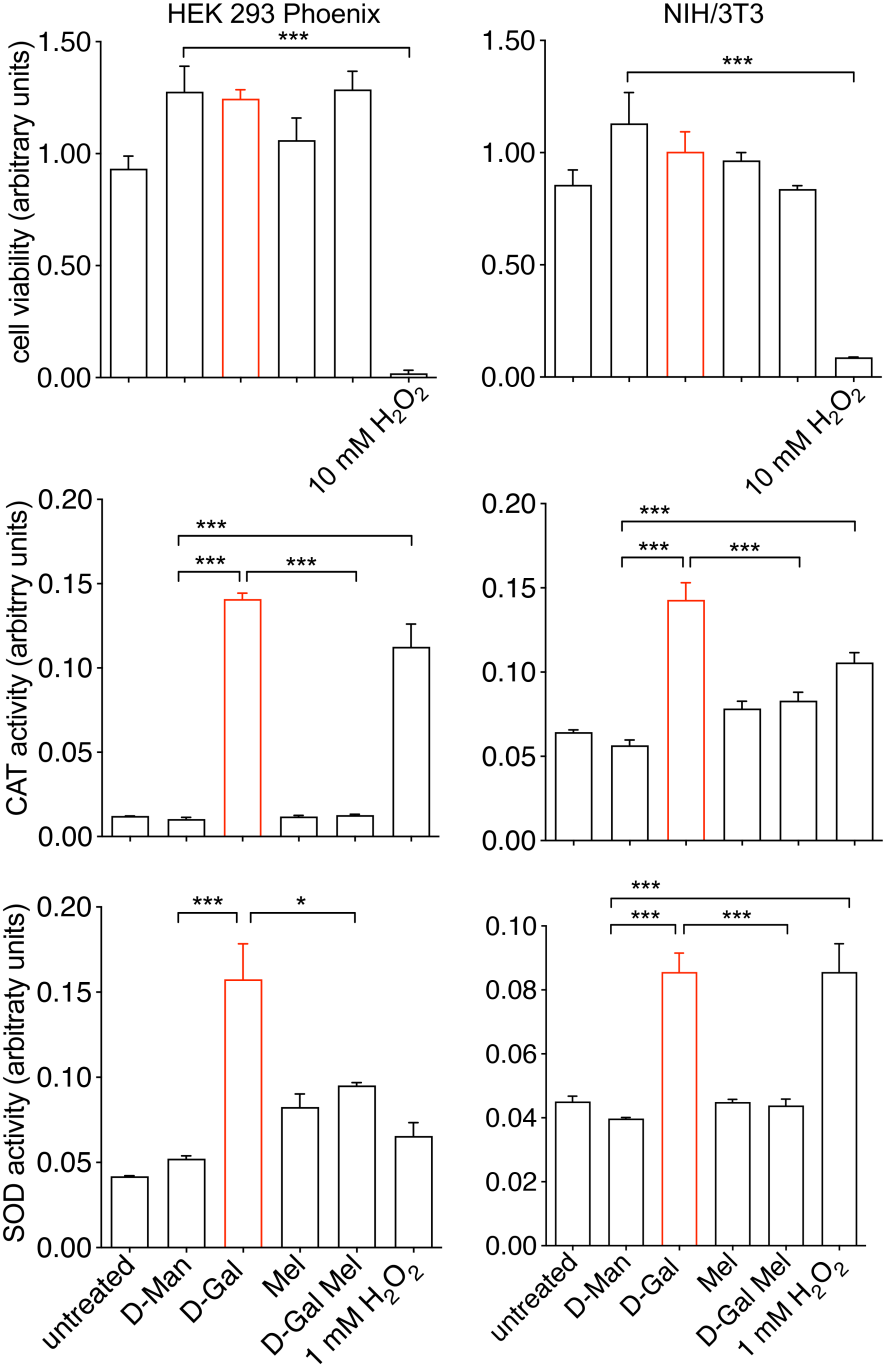
Cell viability, D‐Gal‐induced CAT and SOD activity, and protective effect of melatonin. Cell viability, catalase (CAT), and superoxide dismutase (SOD) activity in HEK 293 Phoenix (left) or NIH/3 T3 cells (right), which were left untreated or exposed to 100 mM D‐Man, D‐Gal, 100 μM melatonin (Mel), or D‐Gal and melatonin for 48 h. Cell treated with 10 or 1 mM H_2_O_2_ for 30 min were included as a reference for conditions of reduced cell viability and elevated oxidative stress, respectively. *p < 0.05, ***p < 0.001, one‐way ANOVA followed by Bonferroni's post hoc test. Data are from 6 (cell viability) or 3 (CAT and SOD activity) biological replicates.

As previously mentioned, D‐Gal leads to oxidative stress via a dual mechanism including H_2_O_2_ generation and inhibition of the mitochondrial function. Increased production of intracellular H_2_O_2_ might lead to the activation of the endogenous antioxidant enzyme CAT. In addition, inhibition of the electron transport chain in the mitochondria leads to the production of superoxide radicals, which might lead to activation of SOD (Bolisetty & Jaimes, 2013). Therefore, the activity of CAT and SOD was measured in our experimental setting. Exposure of cells to D‐Gal for 48 h induced significant activation of CAT as well as SOD, which was consistent with a cellular response triggered by increased oxidative stress, and this activation was effectively blunted by melatonin (Figure 3). In these conditions, no significant effects on cell viability could be observed (Figure 3). Despite a significant alteration of oxidative stress markers (ROS, TBARS, and SH groups), concomitant activation of CAT and SOD most likely protected the cells from excessive damage, thereby preserving cell viability.

Collectively, these findings indicated that significant oxidative stress on cellular proteins and lipids occurred after exposure of cells to D‐Gal, and further support the role of melatonin as a direct free radical scavenger rather than an activator of the endogenous antioxidant system in this context.

### 3.3 Endogenous Kv currents are downregulated in the oxidative stress‐based model of aging

**FIGURE 4:**
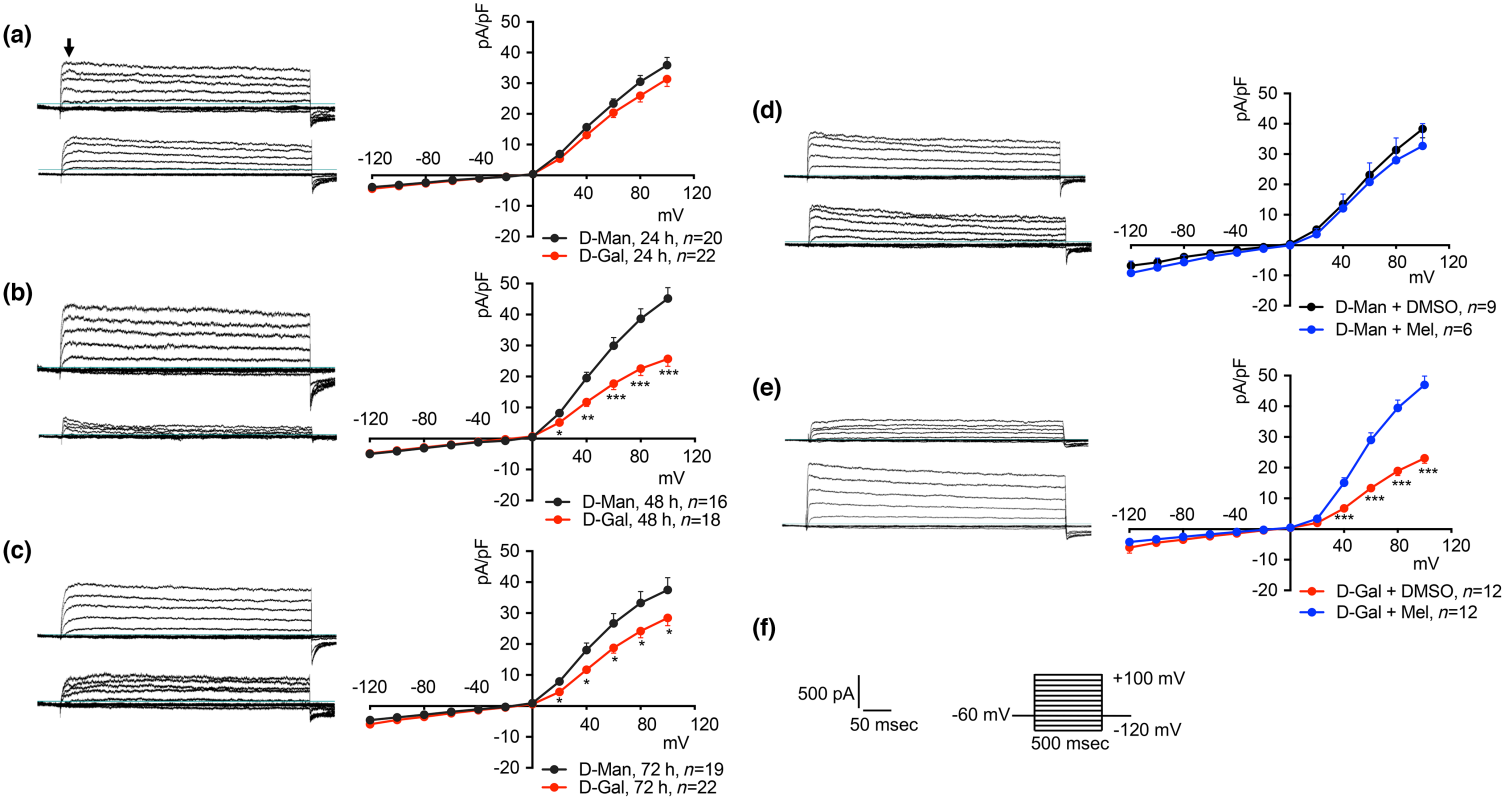
Time‐dependent inhibitory effect of D‐Gal on endogenous Kv3.1 activity and protective effect of melatonin. Original patch‐clamp whole‐cell recordings corresponding to HEK 293 Phoenix cells treated with 100 mM D‐Man (top) or D‐Gal (bottom) and corresponding current density‐to‐voltage relationships obtained after (a) 24 h (b) 48 h, or (c) 72 h of incubation and cells treated for 48 h with (d) 100 mM D‐Man or (e) 100 mM D‐Gal with 0.1% DMSO (top) or 100 μM melatonin (bottom), and corresponding current density‐to‐voltage relationships. Single cells were selected and voltage‐clamped using the whole‐cell patch‐clamp technique. The arrow in (a) indicates the current values taken to calculate the current density and also applies to (b–e). *p < 0.05, ** *p* < 0.01, ***p < 0.001, two‐tailed, unpaired Student's *t*‐test. (*n*) refers to the number of cells. (f) The stimulation protocol consisted of voltage steps from −120 to +100 mV in 20 mV increments from a holding potential of −60 mV. The duration of the voltage steps was 500 ms.

In HEK 293 Phoenix cells, patch‐clamp measurements in whole‐cell configuration with intracellular and extracellular solutions appropriately designed to isolate potassium currents showed a large endogenous outward, rapidly activating, slowly inactivating current with a voltage of activation of −20 mV (Figure 4a). This current is selective for the K^+^ ion, was extensively characterized in our former studies, and was mainly attributed to the Kv3.1a channel (Dossena et al., 2005). Exposure of cells to 100 mM D‐Gal for 48–72 h led to a significant inhibition of this K^+^ current (Figure 4b,c). The current inhibition was not statistically significant after 24 h but reached its maximum extent (44.5%) after 48 h. These observations support the hypothesis of Kv3.1a as a target of oxidative stress. The absence of a significant inhibition after a 24‐h exposure of cells to D‐Gal, when ROS production was maximal (Figure 2a), suggests that the channel protein or its lipid environment are not a direct target of oxidative stress, but rather points to the involvement of targets on a transcriptional, translational, or post‐translational level.

### 3.4 Melatonin restored Kv currents in the oxidative stress‐based model of aging

In a separate set of experiments, HEK 293 Phoenix cells were exposed to 100 mM D‐Man or D‐Gal in the presence or absence of 100 μM melatonin or its vehicle (0.1% DMSO). Melatonin was ineffective in cells exposed to D‐Man, leading to exclude a possible direct effect of melatonin on the channel (Figure 4d). In contrast, melatonin restored Kv currents to control levels in D‐Gal‐treated cells (Figure 4e). These observations support the role of melatonin as an antioxidant rather than a channel activator.

### 3.5 Kv currents are not inhibited by acute oxidative stress

Amino acid residues of cellular proteins, especially cysteine, histidine, methionine, tryptophan, and tyrosine can be relatively easily oxidized, with a profound impact on protein structure and function (Milligan et al., 2003). To test whether Kv channels endogenously expressed in HEK 293 Phoenix cells can be a direct target of oxidative stress, cells were voltage‐clamped and perfused with a strongly pro‐oxidant bath solution containing 1 mM H_2_O_2_ after the establishment of the whole‐cell configuration. In these conditions, Kv currents showed a small, not significant rundown that did not statistically differ from the spontaneous rundown normally observed over 5 min (Figure S2). However, exposure of cells to 10 mM TEA, which is a well‐known inhibitor of voltage‐gated K^+^ channels, completely and rapidly blunted the Kv currents (Figure S2), in agreement with previous findings (Dossena et al., 2005). These observations lead to ruling out Kv channels or their lipid environment as direct targets of oxidative stress. However, small intracellular components, which are lost in the patch‐clamp whole‐cell configuration, could, in principle, be a target of oxidative stress. To test this hypothesis, intact cells were exposed to 1 mM H_2_O_2_ in the cell culture media for 30 min, and the activity of Kv currents was tested afterward. Of note, a 30‐min exposure of cells to 1 mM H_2_O_2_ led to a dramatic elevation of TBARS levels, as well as a significant oxidation of SH groups of cellular proteins (Figure 2b,c), which was accompanied by activation of the endogenous antioxidant enzymes CAT and SOD (Figure 3), denoting a strong pro‐oxidant cellular environment. However, in these conditions, no significant effect on the magnitude of Kv currents was recorded. These findings clearly show that prolonged exposure to oxidative stress is required to affect Kv activity, which is in line with the findings shown in Figure 4.

### 3.6 Kv3.1a is sensitive to oxidative stress and is protected by melatonin

**FIGURE 5:**
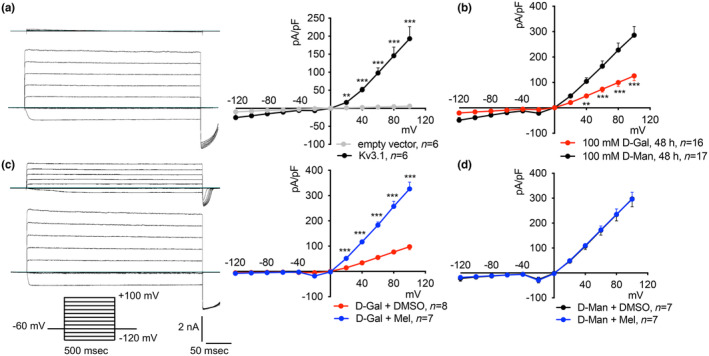
Inhibitory effect of D‐Gal on ectopic Kv3.1 activity and protective effect of melatonin. (a) Original whole‐cell recordings of NIH/3 T3 cells transfected for 48 h with an empty vector (top) or a vector encoding for Kv3.1a (bottom) and corresponding current density‐to‐voltage relationship (right). (b) Current density‐to‐voltage relationship of cells transfected and incubated with 100 mM D‐Man or D‐Gal for 48 h. (c) Original whole‐cell recordings of NIH/3 T3 cells transfected and incubated with 100 mM D‐Gal and 0.1% DMSO (top) or 100 μM melatonin (Mel, bottom) for 48 h and corresponding current density‐to‐voltage relationship (right). (d) Current density‐to‐voltage relationship of NIH/3 T3 cells transfected and incubated with 100 mM D‐Man and 0.1% DMSO or 100 μM melatonin (Mel) for 48 h. Single cells were selected and voltage‐clamped using the whole‐cell patch‐clamp technique. The stimulation protocol in c also applies to a, b, and d. ** *p* < 0.01, ***p < 0.001, two‐tailed, unpaired Student's *t*‐*test*. (*n*) refers to the number of cells.

Being Kv3.1a the main Kv channel expressed in HEK 293 Phoenix cells, it is likely that Kv3.1a is the main determinant of the endogenous Kv current and, consequently, also the main target of oxidative stress. To confirm this hypothesis, a heterologous expression system in NIH/3 T3 cells was set up. Native NIH/3 T3 cells do not express potassium currents detectable in the whole‐cell configuration of patch‐clamp but develop a very large voltage‐dependent current 48 h after transfection with Kv3.1a (Figure 5a). Incubation of cells with 100 mM D‐Gal led to a 56% current inhibition compared to the control condition (100 mM D‐Man, Figure 5b). Importantly, also in these cells, D‐Gal could elicit prominent oxidative stress, as denoted by activation of CAT and SOD, with no influence on cell viability (Figure 3). Also, cellular ROS elevation was significant after a 6‐h exposure to D‐Gal, reached the maximum extent after 24–48 h, and was reverted by melatonin (Remigante et al., 2023). Melatonin could restore Kv3.1a currents to control values in transfected NIH/3T3 cells exposed to D‐Gal (Figure 5c), while it was ineffective on Kv3.1a currents measured in cells exposed to D‐Man (Figure 5d). These findings confirm that Kv3.1 can be targeted by chronic oxidative stress and that melatonin can protect Kv3.1a activity with no direct effect on the Kv3.1‐induced current.

### 3.7 Kv3.1 transcript and protein levels are unaffected in the oxidative stress‐based model of aging

**FIGURE 6:**
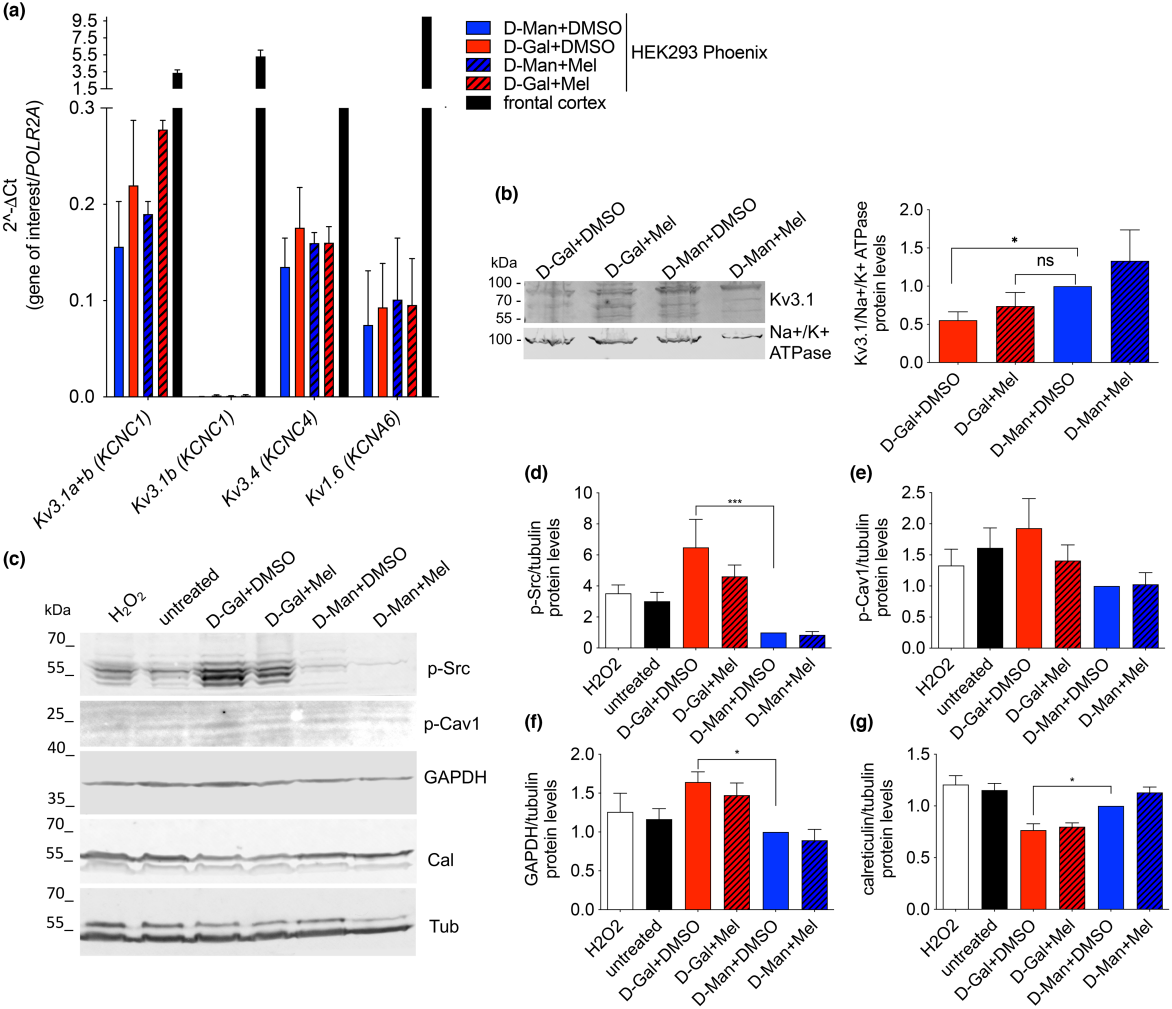
Verification of possible D‐Gal‐induced changes in the Kv transcripts and Kv3.1 plasma membrane protein abundance and exploration of intracellular events elicited by exposure to D‐Gal. Native HEK 293 Phoenix cells were treated with 100 mM D‐Man or D‐Gal in the presence of 100 μM melatonin (Mel) or its vehicle (0.1% DMSO) for 48 h. (a) mRNA levels of the indicated Kv genes. Human frontal cortex cDNA was included to confirm primer efficiency. (b) Original blot (left) and densitometry (right) of Kv3.1a protein levels in plasma membrane fractions. (c) Original blots and densitometry of (d), p‐Src, (e), p‐Cav1, (f) GAPDH, and (g) calreticulin protein levels in total protein extracts. Individual densitometry values have been normalized for those of the housekeeping proteins Na^+^/K^+^‐ATPase (plasma membrane fractions) or tubulin (total protein fractions). Each data set has been normalized to D‐Man+DMSO from the same gel. Data from 4 (plasma membrane fractions) or 6–8 (total protein fractions) independent biological replicates have been analyzed with the one‐way ANOVA with Dunnet's multiple comparison post‐test. n.s., not statistically significant, *p < 0.05, ***p < 0.001.

To test whether the reduction in Kv current magnitude observed after exposure to D‐Gal could arise from a reduction in the levels of Kv transcripts and/or protein, the abundance of Kv3.1, Kv3.4, Kv1.6 transcript were measured by RT‐qPCR in HEK293 Phoenix cells exposed to D‐Gal or D‐Man in the presence of melatonin or its vehicle. No significant variation in Kv transcript abundance among the different conditions was found in these cells, indicating that D‐Gal‐induced oxidative stress most likely did not affect Kv mRNA transcription or stability (Figure 6a). Also, a possible effect of D‐Gal was tested on the endogenous Kv3.1a total protein abundance (Figure S3A), as well as on Kv3.1a ectopically expressed in HEK293 Phoenix cells (Figure S3B). No significant differences were found among the conditions tested, indicating that an effect of oxidative stress on Kv3.1 protein translation and/or stability is unlikely.

### 3.8 Oxidative stress affects Kv3.1a protein abundance at the plasma membrane

Reduced current density with no direct channel inhibition can result from reduced channel units in the plasma membrane consequent to altered protein trafficking (either reduced anterograde translocation of the protein channel from the endoplasmic reticulum through the Golgi to the plasma membrane or increased retrograde translocation via internalization/endocytosis). To test whether altered trafficking to the plasma membrane might contribute to the reduction of Kv3.1 activity observed after exposure to D‐Gal, the abundance of the Kv3.1a protein was tested in plasma membrane‐enriched fractions from native HEK293 Phoenix cells exposed to D‐Gal or D‐Man in the presence of melatonin or its vehicle (Figure 6b). Exposure of cells to D‐Gal for 48 h significantly reduced (44.5%) the Kv3.1a protein abundance in the plasma membrane fraction compared to cells exposed to D‐Man, suggesting an altered trafficking of Kv3.1 to the cell surface.

### 3.9 Reduced plasma membrane Kv3.1a abundance is associated with Src phosphorylation as well as metabolic and endoplasmic reticulum stress

As the tyrosine‐protein kinase Src controls the subcellular localization of Kv3.1b (Bae et al., 2014) and oxidative stress is known to activate Src and caveolin 1 (Cav1) (Hoshi et al., 2020), we verified whether these events occurred in our setting. Exposure of cells to D‐Gal significantly increased the phosphorylation levels of the Src family of protein kinases compared to control cells treated with D‐Man (Figure 6c,d). The levels of phosphorylated Cav1 almost doubled in D‐Gal‐treated cells, however, this variation did not reach statistical significance (Figure 6e). Also, significantly elevated levels of the glycolytic enzyme glyceraldehyde 3‐phosphate dehydrogenase (GAPDH, Figure 6f), and reduced expression of the endoplasmic reticulum‐resident protein calreticulin (Figure 6g) were observed in D‐Gal‐treated cells, indicating metabolic reprogramming and endoplasmic reticulum stress, respectively.

## 4 DISCUSSION

Owing to the fundamental role of Kv3.1 in permitting the activity of inhibitory interneurons, it is not surprising that a loss of function of Kv3.1 might lead to neuronal hyperexcitability in the brain. Indeed, a recurrent pathogenic heterozygous sequence variant in *KCNC1* (gene MIM number 176258) was identified as a cause of childhood‐onset progressive myoclonic epilepsy variability associated with ataxia, tonic–clonic seizures, learning disabilities, and mild cognitive decline. The authors called this phenotypic spectrum “myoclonic epilepsy with ataxia due to potassium channel mutation” (MEAK), also designated “progressive myoclonic epilepsy 7” (EPM7, phenotype MIM number 616187). Functional testing of the corresponding protein variant (p.Arg320His) showed a loss of function with a dominant negative effect (Muona et al., 2015). With the widespread use of next‐generation sequencing techniques, several *KCNC1* pathogenic variants were identified in patients with infantile‐onset developmental and epileptic encephalopathy (DEE), developmental encephalopathy without seizures, non‐progressive myoclonus without intellectual disability, and variants of MEAK with radiologic abnormalities of the brain cortex on the MRI (Cameron et al., 2019). It is therefore tempting to speculate that also loss of function of Kv3.1 independent of genetic mutation might contribute to epileptogenesis and epilepsy in humans.

After the onset of hearing, which corresponds to postnatal day P12 in mice and embryonic week EW27 in humans, Kv3.1 becomes abundantly expressed along the entire ascending auditory pathway, starting from the rapidly firing spiral ganglion neurons to the parvalbumin‐positive interneurons of the cochlear nucleus projecting onto the lateral and medial superior olive and medial nucleus of the trapezoid body (MNTB), projections from these nuclei to the inferior colliculus, from the inferior colliculus to the medial genicular body, and finally to the auditory cortex (Peixoto Pinheiro et al., 2021). Principal neurons that fire at very rapid rates within the auditory pathway require the expression of Kv3.1 for rapid repolarization of action potentials during sound‐induced activity. Accordingly, MNTB neurons from Kv3.1 knockout mice exhibited complete loss of action potential generation following stimulation at high frequencies (Macica et al., 2003). Importantly, a decline in Kv3.1 expression was found in the MNTB of C57BL/6 mice, which develop early hearing loss after 6 months of age (von Hehn et al., 2004), in the auditory brainstem of middle‐aged and older CBA/CaJ mice (Zettel et al., 2007), and in the posterior ventral cochlear nucleus of aged rats (Jung et al., 2005). It is therefore conceivable that the loss of function of Kv3.1 might contribute to age‐related hearing loss in humans.

Although oxidative stress plays a fundamental role in determining epilepsy, epileptogenesis, and age‐related hearing loss, the molecular targets that are dysregulated during the early pathophysiological processes are far from being unequivocally established. The elevated oxidative status in patients with epilepsy is independent of the exposure to antiepileptic drugs, denoting that although current therapies can control neuronal hyperexcitability, they might be poorly effective in offering neuroprotection and treating epileptogenesis (Borowicz‐Reutt & Czuczwar, 2020). Epileptogenesis, which is the seizure‐free period where a cascade of events leading to spontaneous recurrent seizures occurs, offers a unique window of opportunity for targeted interventions to prevent or attenuate seizure development.

Oxidative stress, altered levels of antioxidant enzymes, and mitochondrial dysfunction are found in various models of age‐related hearing loss; similarly, premature hearing loss was observed in mouse models with altered endogenous antioxidant capacity. In animal studies, antioxidant supplementation often showed beneficial effects, with variable results depending on the specific antioxidant(s), the timing of administration, and the duration of treatment (Fujimoto & Yamasoba, 2014). These observations highlight that the identification of early targets of oxidative stress and their protection prior to irreversible damage occurring is of the outmost importance.

The data presented here show a significant loss of function of both endogenous and ectopically expressed Kv3.1a in cellular models of D‐Gal‐induced aging (Figures 4b,c and 5b). These alterations were paralleled by increased ROS production, lipid peroxidation, depletion of SH groups of cellular proteins (Figure 2), and upregulation of the endogenous CAT and SOD activities (Figure 3), all denoting prominent oxidative stress in these cells. Despite that, no reduction in cellular viability was observed, indicating that gross dysregulation of the cell homeostasis did not occur. Based on this, we suggest that Kv3.1a might represent an early and specific molecular target of oxidative stress that could be amenable to pharmacological protection.

Direct exposure of cells to strong pro‐oxidant conditions (1 mM H_2_O_2_ for 5–30 min) failed to reproduce the Kv3.1a current inhibition observed in the D‐Gal model (Figure S2). Of note, exposure to 1 mM H_2_O_2_ for 30 min led to abundant SH group oxidation of cellular proteins as well as TBARS production (Figure 2), denoting significant oxidative stress. Therefore, we conclude that the direct oxidation of amino acid side chains of the channel protein or membrane lipid peroxidation probably did not account for the Kv3.1 current inhibition observed in the D‐Gal model. Also, the temporal delay occurring between the ROS peak production (24 h, Figure 2) and the greatest current inhibition (48 h, Figure 4b) denotes that intracellular events are most likely involved. However, no changes in *KCNC1* transcript or protein levels were observed (Figure 6a and Figure S3), leading to exclude a reduction in gene transcription, mRNA stability, or an increased protein degradation.

In principle, current magnitude and biophysical properties can be modulated by phosphorylation of the channel. As mentioned above, Kv3.1b phosphorylation at Ser503 by PKC is capable of dramatically decreasing the Kv3.1b current density (Macica et al., 2003). However, this amino acid residue is missing in Kv3.1a and, therefore, cannot account for the current inhibition that we have observed. Potential CK2 phosphorylation sites in the Kv3.1 channel proteins are found in both the C and N terminus, the S5–S6 linker, and the intracellular S4/S5 linker. However, abundant CK2 phosphorylation in basal conditions controls the constitutive biophysical properties of the channel, and dephosphorylation increases the current magnitude while shifting the activation potential to values more negative of at least 20 mV (Macica & Kaczmarek, 2001). Therefore, we exclude an involvement of CK2‐mediated phosphorylation in our setting.

The subcellular localization of Kv3.1b is controlled by the tyrosine‐protein kinase Src. In HEK293 and COS‐7 cells, a constitutively active form of Src inhibited Kv3.1b trafficking to the plasma membrane, induced a dramatic redistribution of the protein channel to the endoplasmic reticulum, and resulted in a significant decrease in the current density (Bae et al., 2014). Notably, oxidative stress is known to activate Src, and caveolin 1 is involved in the downstream pathway (Hoshi et al., 2020). Whether Src directly phosphorylates Kv3.1b and also affects the function of Kv3.1a was unknown. In our D‐Gal model, a reduction of Kv3.1a protein abundance in the plasma membrane‐enriched fraction was observed (Figure 6b). The extent of Kv3.1a protein abundance reduction in the plasma membrane fraction of HEK 293 Phoenix cells (44.5%) following exposure to 100 mM D‐Gal for 48 h is identical to the extent of endogenous Kv current reduction observed in these same cells in the same conditions (44.5%, Figure 4b), which suggests a causative link between these two phenomena. We tested whether phosphorylation events elicited by oxidative stress might have reduced the Kv3.1a trafficking to the plasma membrane and therefore attenuated the Kv3.1a‐elicited current. As hyperosmolarity is known to inhibit Src (Kapus et al., 1999), D‐Man‐treated cells used as a control also accounted for the effect of osmolarity. We have found prominent phosphorylation, and hence activation, of the Src family of kinases in cells treated with D‐Gal compared to D‐Man (Figure 6c,d). Src activation and subsequent phosphorylation of Cav1 and/or other targets might have led to Kv3.1a internalization, as reported for P‐glycoprotein (Hoshi et al., 2020).

We also found an increased expression of the glycolytic enzyme GAPDH in cells exposed to D‐Gal for 48 h compared to control cells incubated in the presence of the non‐metabolizable D‐Man (Figure 6f). This might have reflected metabolic activation, as D‐Gal can enter glycolysis after being metabolized to galactose‐1‐phosphate by galactokinase, which might have contributed to oxidative stress. In addition, GAPDH is a well‐known redox sensor, as it contains an active‐site cysteine residue undergoing oxidation in response to oxidative stress, leading to rapid inactivation of the enzyme and rerouting the cell metabolism from glycolysis to the pentose phosphate pathway (Talwar et al., 2023). Thus, GAPDH upregulation may also have resulted from a physiological adaptation to the inactivation of this enzyme during oxidative stress. In addition to these metabolic functions, GAPDH is now recognized as a multifunctional protein involved in transcriptional and posttranscriptional gene regulation and intracellular membrane trafficking, and undergoes nuclear translocation and aggregation under oxidative stress, leading to mitochondrial dysfunction (Nakajima et al., 2017). Protein aggregation is well‐known to lead to endoplasmic reticulum stress. Interestingly, in D‐Gal‐treated cells, we have documented a reduction in the expression of the endoplasmic reticulum‐resident protein calreticulin (Figure 6g), which is a hallmark of endoplasmic reticulum stress (Kuang et al., 2014). Therefore, phosphorylation events involving Src and caveolins as well as metabolic and endoplasmic reticulum stress might all have contributed to a decreased Kv3.1a trafficking to the plasma membrane.

D‐Gal‐treated experimental animals have been widely utilized as models of aging and age‐related conditions, including neurodegeneration and neuroinflammation (Shwe et al., 2018). In light of our findings, it would be important to verify whether these animals are more prone to develop seizures. Concerning hearing loss, cochlear structures, and especially the stria vascularis and cochlear ribbon synapsis between hair cells and spiral ganglion neurons were altered in D‐Gal mice and rats (Du et al., 2020; Peng et al., 2023). Oxidative stress, mitochondrial DNA damage, and neuronal apoptosis have also been observed at retrocochlear sites including the auditory cortex, inferior colliculus, and cochlear nucleus (Chen et al., 2010). Future studies should address whether an alteration of Kv3.1 membrane abundance can be observed at these sites prior to neuronal apoptosis occurring.

Importantly, the Kv3.1 loss of function observed in our system was reverted by pharmacological concentrations of melatonin. This occurred on both the endogenous and the ectopically expressed current (Figures 4 and 5). At the same time, melatonin blunted ROS and TBARS generation, protected SH groups from oxidation, and prevented the activation of the endogenous antioxidant enzymes (Figures 2 and 3).

The oxidative stress theory of aging postulates that accumulation of oxidative damage over time leads to the progressive decline of tissue and organ function that characterizes older age. At the same time, oxidative stress is regarded as the primary cause or the secondary contributor to several age‐related conditions, including atherosclerosis, chronic obstructive pulmonary disease, cancer, diabetes, cardiovascular diseases, Alzheimer's disease, age‐related hearing loss and epilepsy (Forman & Zhang, 2021). The fundamental contribution of oxidative stress to the pathogenesis of epilepsy and age‐related hearing loss implies that antioxidants, including vitamins and nutrients, might play a protective role in this context (Borowicz‐Reutt & Czuczwar, 2020; Madireddy & Madireddy, 2023). Accordingly, melatonin has been shown to have neuroprotective effects against epilepsy in various animal models and in humans. For instance, melatonin showed beneficial effects in animal models of iron‐induced (Kabuto et al., 1998), penicillin‐induced (Yildirim & Marangoz, 2006), pentylenetetrazol‐induced (Solmaz et al., 2009), and kainate‐induced seizures (Tchekalarova et al., 2013). Recent clinical trials support the use of melatonin as an add‐on to reduce seizure frequency or severity (Maghbooli et al., 2023; Verma et al., 2021).

Concerning hearing loss, melatonin showed beneficial effects in cisplatin‐induced hearing loss in rats (Demir et al., 2015) and noise‐induced hearing loss in guinea pigs (Karlidag et al., 2002), had otoprotective effects and ameliorated age‐related hearing loss in C57BL/6J mice and rats (Seidman, 2000; Serra et al., 2020). However, to our knowledge, the possible pharmacological use of melatonin in patients to prevent or delay the progression of age‐related hearing loss, or other forms of oxidative stress‐related hearing loss such as cisplatin‐induced hearing loss, was not explored.

To conclude, here we report on a novel Kv3.1 channel modulation occurring during oxidative stress. We suggest that oxidative stress‐dependent inhibition of Kv3.1 in principal neurons of the auditory pathway and GABAergic inhibitory interneurons in the brain might contribute to age‐related hearing loss and epilepsy during aging. Melatonin might play a protective role in this context.

## AUTHOR CONTRIBUTIONS

*Conceptualization*: Sara Spinelli, Alessia Remigante, Angela Marino, Rossana Morabito, Silvia Dossena; *Methodology*: Giuseppe Legname, Angela Marino, Rossana Morabito, Silvia Dossena; *Formal analysis and investigation*: Sara Spinelli, Raffaella Liuni, Gianluca Mantegna, Silvia Dossena; *Writing*—*original draft preparation*: Silvia Dossena; *Writing*—*review and editing*: all authors; *Visualization*: Alessia Remigante, Sara Spinelli, Silvia Dossena; *Supervision*: Angela Marino, Rossana Morabito, Silvia Dossena. Sara Spinelli and Alessia Remigante contributed equally and shared the first authorship.

## FUNDING INFORMATION

A.R. was supported by the Programma Operativo Nazionale (PON) Ricerca e Innovazione 2014‐2020, D.M. 1062/2021. The research was funded by Progetti di Rilevante Interesse Nazionale (PRIN) 2022YRBE8B to Angela Marino and projects 2022‐Iif‐004‐DOSSENA and FIZ RM&NT Talent Pool SR 042023 to Silvia Dossena.

## CONFLICT OF INTEREST STATEMENT

The authors declare no conflicts of interest.

## Supporting information


Data S1:

## Data Availability

The data that support the findings of this study are available from the corresponding author upon reasonable request.
