# Supplementary material for: Oxidative stress‐related cellular aging causes dysfunction of the Kv3.1/KCNC1 channel reverted by melatonin
Source: Aging Cell. 2024 May 9;23(8):e14185. doi: 10.1111/acel.14185 (PMC11320344; doi:10.1111/acel.14185)
Supplement: Supplementary file 1 — Data S1: [file ACEL-23-e14185-s001.docx]

**Supplemental Information**

**Supplemental Methods**

**Cell viability assay**

Cell viability was evaluated by the CellTiter 96® AQueous One Solution Cell Proliferation Assay system (Promega, Madison, WI, USA), according to the manufacturer’s instructions. The CellTiter 96® AQueous One Solution Reagent contains a tetrazolium compound (3-(4,5-dimethylthiazol-2-yl)-5-(3-carboxymethoxyphenyl)-2-(4-sulfophenyl)-2H-tetrazolium, inner salt) that is bioreduced by cells into a colored soluble formazan product. The amount of formazan produced is directly proportional to the number of living cells in the culture. Briefly, cells were seeded into 96-well plates with a density of 1 x 10^6^ cells/well. After exposure to the test compounds, cells were incubated with 20 μL CellTiter 96® AQueous One Solution Reagent for 3 hours in a humidified 5% CO_2_ atmosphere. The formation of formazan was determined at 490 nm absorbance (Victor *X3* multiplate reader, Perkin Elmer, Waltham, MA, USA) after subtracting the background absorbance. Six replicate wells for each condition were measured in each experiment.

**Determination of thiobarbituric acid reactive substances**

Thiobarbituric acid reactive substances (TBARS) levels were evaluated as formerly described (Mendanha et al., 2012) with minor modifications. TBARS are produced by the reaction between thiobarbituric acid (TBA) and malondialdehyde (MDA), which is the final product of lipid peroxidation. Briefly, cells were seeded in 150 mm diameter Petri dishes, grown to ~80% confluence, and exposed to the test compounds. After each treatment, cell pellets were suspended in an isotonic solution (in mM: NaCl 150, 4-(2-hydroxyethyl)-1-piperazineethanesulfonic acid (HEPES) 5, glucose 5, pH 7.4, 300 mOsm/Kg_H2O_), treated with 10% (w/v) trichloroacetic acid and centrifuged (Neya 16R, 3000 *xg*, 10 min). TBA (1% in 0.05 M NaOH, 0.5 ml) was added to the supernatant and the mixture was heated at 95 °C for 2 hours. TBARS levels were obtained by subtracting 20% of the absorbance at 453 nm from the absorbance at 532 nm (Onda Spectrophotometer, UV-21). Results are indicated as µM TBARS (molar extinction coefficient: 1.56 x 10^5^ M^-1^ cm^-1^).

**Determination of total sulfhydryl group content**

Total sulfhydryl (-SH) group content was evaluated as formerly described (Aksenov & Markesbery, 2001), with minor modifications. Briefly, cells were seeded in 150 mm diameter Petri dishes, grown to ~80% confluence, and exposed to the test compounds. Cell pellets were lysed on ice in 1 mL of buffer containing 20 mM Tris-HCl, 150 mM NaCl, 1 mM EDTA, 0.1% NP40, and protease inhibitor cocktail (Roche, Basel, Switzerland). Subsequently, cellular debris was discarded by centrifugation at 16000 *xg* for 30 min at 4 °C and the supernatant was collected. Samples were incubated with 1% SDS for 20 min on ice and centrifuged (Neya 16R, 13000 *xg*, 10 min, 4 °C). To start the reaction, a 10 µL aliquot of the supernatant was added to 90 µL 5,5'-dithio-bis-[2-nitrobenzoic acid] (DTNB, 1 mM), and the samples were incubated at 25 °C for 30 min protected from light. After incubation, sample absorbance was measured at 412 nm (Onda spectrophotometer, UV-21) and 3-thio-2-nitro-benzoic acid (TNB) levels were detected after subtraction of blank absorbance (samples containing DTNB only). Results are reported as µM TNB.

**Determination of intracellular reactive oxygen species**

Total intracellular ROS levels were evaluated with the 2’,7’-dichlorofluorescein diacetate indicator (H2DCFDA, D6883, Sigma-Aldrich) (Liu et al., 2017). The non-fluorescent compound H2DCFDA is first de-acetylated and then oxidized by ROS to the fluorescent compound dichlorofluorescein (DCF). Cells were seeded into 96-well plates to a density of 1 x 10^6^ cells/well. After 24 hours, cells were exposed to the test compounds for 3-72 hours. As a positive control, cells were treated with 1 mM H_2_O_2_ for 30 min. After each treatment, the incubation medium was discarded and cells were washed in phosphate-buffered saline (PBS). Subsequently, cells were incubated with 50 µL of 20 µM H2DCFDA diluted in PBS for 45 min in a humidified 5% CO_2_ atmosphere at 37 °C. Finally, cells were washed with PBS. Fluorescence measurements were performed with a microplate reader (Victor *X3*, Perkin Elmer, excitation wavelengths: 485 nm, emission wavelength: 535 nm), after subtracting the background fluorescence. Six replicate wells for each condition were measured in each experiment.

**Catalase activity assay**

Cells were seeded into 6-well plates to a density of 1 x 10^6^ cells/well. After 24 hours, the cells were exposed to the test compounds. A subset of cells was treated with 1 mM H_2_O_2_ for 30 min. After each treatment, the incubation medium was discarded, and cells were washed in PBS. Catalase (CAT) activity was evaluated by the Catalase Assay Kit (MAK381, Sigma-Aldrich), according to the manufacturer’s instructions. The absorbance of samples was determined at 595 nm wavelength (Victor *X3* multi-plate reader, Perkin Elmer) after subtracting the background absorbance. Three biological replicates were used to obtain measures of CAT activity.

**Superoxide dismutase activity assay**

Cells were seeded into 6-well plates to a density of 1 x 10^6^ cells/well. After 24 hours, the cells were exposed to the test compounds. A subset of cells was treated with 1 mm H_2_O_2_ for 30 min. After each treatment, the incubation medium was discarded, and cells were washed in PBS. Superoxide dismutase (SOD) activity was evaluated by the Superoxide Dismutase (SOD) Activity Assay Kit (CS0009, Sigma-Aldrich), according to the manufacturer’s instructions. The absorbance of samples was determined at 450 nm wavelength (Victor *X3* multi-plate reader, Perkin Elmer) after subtracting the background absorbance. Three biological replicates were used to obtain measures of SOD activity.

**Patch-clamp experiments**

Cells for electrophysiology were seeded on glass coverslips (diameter, 10 mm) contained in 30 mm diameter Petri dishes and grown overnight. Single cells were selected by phase contrast or fluorescence microscopy as appropriate and voltage-clamped using the whole-cell patch-clamp technique as previously described, with minor modifications (Costa et al., 2018; Costa et al., 2020; Dossena et al., 2011; Dossena et al., 2005; Gandini et al., 2008; Garavaglia et al., 2008; Guizzardi et al., 2006; Kossler et al., 2012; Li et al., 2014; Meyer et al., 2004; Morabito et al., 2017; Tamma et al., 2011). The pipette-filling solution contained (in mM): potassium gluconate 115, MgCl_2_ 5, ethylene glycol-bis(β-aminoethyl ether)-N,N,N',N'-tetraacetic acid (EGTA) 5, HEPES 5, ATP magnesium salt 5, mannitol 50, 300 mOsm/Kg_H2O_, pH 7.2. To obtain the whole-cell configuration, 167 μg/mL nystatin was added to the pipette-filling solution from a 50 mg/mL stock prepared in dimethyl sulfoxide (DMSO) (Horn, 1991). The bath solution had the following composition (in mM): potassium gluconate 115, NaCl 5.4, CaCl_2_ 1.8, MgCl_2_ 0.5, HEPES 5, glucose 5, mannitol 50, 300 mOsm/Kg_H2O_, pH 7.4. With these solutions, the resistance of the glass pipettes was 3 to 8 MΩ. The bath was connected to the reference electrode via a 100 mM KCl, 4% agar bridge. For perfusion experiments, a fast exchange of the bath solution was accomplished using a perfusion system with a flow rate of 5 ml/min and a bath volume of ∼300 μl. To establish the current-to-voltage relationship, the cells were stimulated with step pulses of 500 ms duration from -120 mV to +100 mV in 20 mV increments from a holding potential of -60 mV.

**Western Blot**

Total (≥20 μg) or plasma membrane protein extracts were electrophoresed with constant voltage (120 V) on SDS-PAGE gels (10 or 12%). Proteins were then transferred for 2 hours onto polyvinylidene fluoride (PVDF) membranes with constant voltage (75 V). The membranes were blocked for 1 hour at room temperature in 5% w/v nonfat dry milk diluted in Tris-buffered saline containing 0.1% Tween 20 (TBST), incubated overnight at 4 °C with primary antibodies diluted in TBST and 5% nonfat dry milk, washed 3 times in TBST, incubated for 1 hour at room temperature with the secondary antibodies diluted in TBST and 5% nonfat dry milk, washed again and imaged. For Western blot on phosphorylated proteins, 5% nonfat dry milk was substituted for 5% w/v BSA. Immunocomplexes were visualized using the ODYSSEY infrared imaging system (LI-COR, Lincoln, NE, USA). The rabbit polyclonal anti-Kv3.1 antibodies (PA5-42894, 1:400 dilution, and PA1-12659, 1:500 dilution) were from Invitrogen (Waltham, MA, USA). The rabbit polyclonal phospho-Src family (Tyr416) and phospho-Caveolin-1 (Tyr14) (#2101 and #3251, both 1:1000) antibodies were from Cell Signaling Technology (Danvers, MA, USA). The mouse anti-calregulin (sc373863, 1:100) was from Santa Cruz Biotechnology (Santa Cruz, Dallas, TX, USA). The goat anti-GAPDH antibody (PLA0302, 1:1000 dilution) was from Sigma-Aldrich, the mouse monoclonal anti-Na^+^/K^+^-ATPase (05-369, 1:5000 dilution) and anti-tubulin (05-829, 1:1000) were from Millipore (Burlington, MA, USA). The goat anti-rabbit (926-32211), goat anti-mouse (926-32210), and donkey anti-goat (926-32214) IRDye 800 CW secondary antibodies (all diluted 1:20,000) were from LI-COR. The Kv3.1 signal was normalized for the signal of the housekeeping proteins GAPDH or tubulin (total proteins) or Na^+^/K^+^-ATPase (plasma membrane proteins). Densitometry was performed with ImageJ 1.53t software (Wayne Rasband, NIH, MD, USA).

**Supplemental tables**

**Supplemental Table S1:** Characteristics of the donors of human brain samples used in this study.

| **Subject** | **Age (years)** | **Sex** | **Cause of death** | **Neurological signs** |
| --- | --- | --- | --- | --- |
| #19 | 62 | M | Ischemic heart disease  Coronary artery atheroma | No significant abnormalities |
| #20 | 57 | M | Ischemic heart disease  Coronary artery atherosclerosis | No significant abnormalities |
| #23 | 58 | M | Coronary artery thrombosis  Coronary artery atherosclerosis | No significant abnormalities |

**Supplemental Table S2:** Sequence of primers for RT-qPCR.

| **Gene** | **Protein** | **Exon location** | **Primer sequence** | |
| --- | --- | --- | --- | --- |
| *KCNC1* | **Kv 3.1 a+b** | 1-2 | Primer 1 | 5’-CCGTACTCGTCCCGCTA-3’ |
|  |  |  | Primer 2 | 5’-TCTCGATCTCCGTCTTGTTCA-3’ |
| *KCNC1* | **Kv 3.1a** | 2 | Primer 1 | 5’-CTGGGCTGTTCGTCTATCTATG-3’ |
|  |  |  | Primer 2 | 5’-CCCTCGTTGGTTTCTCTTTCT-3’ |
| *KCNC1* | **Kv 3.1b** | 3-4 | Primer 1 | 5’- CCCTGCTTCCTCTTATCAACC-3’ |
|  |  |  | Primer 2 | 5’- CCTCTGTCGGCATATACTTAGC-3’ |
| *KCNA1* | **Kv 1.1** | 1-2 | Primer 1 | 5’-GGCTCTCCGCTGACTCA-3’ |
|  |  |  | Primer 2 | 5’-ACCCTCTCTGCTTCTCCTC-3’ |
| *KCNA2* | **Kv 1.2** | 2-3 | Primer 1 | 5’-CGAGTGAGAAGACGTGATGAG-3’ |
|  |  |  | Primer 2 | 5’-TCTGAGAGCTGGAGAGACAG-3’ |
| *KCNA3* | **Kv 1.3** | 1-1 | Primer 1 | 5’-GATCTCTCTGTGCCATCGC-3’ |
|  |  |  | Primer 2 | 5’-ATTGCTCTTCCCCTTCTGTC-3’ |
| *KCNA4* | **Kv 1.4** | 1-2 | Primer 1 | 5’-TCATTCTTCCCCTCCTCCAG-3’ |
|  |  |  | Primer 2 | 5’-GCAGAGCATTCTTCAGCCA-3’ |
| *KCNA6* | **Kv 1.6** | 1-2 | Primer 1 | 5’-AGCTCGCCTTCTTTGCAG-3’ |
|  |  |  | Primer 2 | 5’-GCTGCTGTCAGAATTCTATGC-3’ |
| *KCNC3* | **Kv 3.3** | 4-5 | Primer 1 | 5’-GATATCCCCCTAGTGGACGAA-3’ |
|  |  |  | Primer 2 | 5’-CCATTCCCAATTGCTAACCTG-3’ |
| *KCNC4* | **Kv 3.4** | 1-2 | Primer 1 | 5’-TCTTCGAGGATCCCTACTCC-3’ |
|  |  |  | Primer 2 | 5’-GCAGAAAGTGGTGATGGAGA-3’ |
| *KCND1* | **Kv 4.1** | 1-2 | Primer 1 | 5’-ACCATTGTCACCATGACCAC-3’ |
|  |  |  | Primer 2 | 5’-AGATGCGGCTAAAGTTGGAC-3’ |

**Supplemental Figures**

**Supplemental Figure S1. Expression profile of Kv channels in human adult brain samples.** Transcript levels of Kv3.1 measured by qRT-PCR in 3 unrelated human subjects (Table S1) with primers specific for the Kv3.1a, Kv3.1b, or both transcripts (illustrated in Figure 1B), and normalized to those of the housekeeping transcript *POLR2A*. Data are from technical duplicates.

**Supplemental Figure S2. Effect of short and long-term exposure to pro-oxidants on Kv activity.** Current density-to-voltage relationship of HEK 293 Phoenix cells before and after perfusion with **A**, the unmodified bath solution, or the bath solution containing **B**, 10 mM TEA or **C**, 1 mM H_2_O_2_. **D**, Cells were incubated with 1 mM H_2_O_2_ in the cell culture medium for 30 minutes and then voltage-clamped. **p<0.01, two-tailed, unpaired Student’s t-test. (n) refers to the number of cells. The voltage protocol consisted of voltage steps from -120 to +100 mV in +20 mV increments from a holding potential of -60 mV. The duration of the voltage steps was 500 ms.

**Supplemental Figure S3. Verification of possible D-Gal-induced changes on the Kv3.1 total protein levels. A,** original blot (left) and densitometry (right) of Kv3.1 protein levels in native (untransfected) HEK 293 Phoenix cells treated with 100 mM D-Man or D-Gal in the presence of 100 µM melatonin (Mel) or its vehicle (0.1% DMSO) for 48 hours. Untreated transfected and untransfected cells were also included for reference. **B,** original blot (left) and densitometry (right) of Kv3.1 protein levels in HEK 293 Phoenix cells transfected with Kv3.1a and treated with 100 mM D-Man or D-Gal in the presence of 100 µM melatonin (Mel) or its vehicle (0.1% DMSO) for 48 hours. Untreated native (untransfected) cells were included for reference. Individual densitometry values have been normalized for those of the housekeeping protein GAPDH. Each data set has been normalized to D-Man+DMSO from the same gel. Data from 3 independent biological replicates have been analyzed with the one-way ANOVA with Bonferroni´s multiple comparison post-test. No significant differences between data sets were found.

**Supplemental References**

Aksenov, M. Y., & Markesbery, W. R. (2001). Changes in thiol content and expression of glutathione redox system genes in the hippocampus and cerebellum in Alzheimer's disease. Neurosci Lett, 302(2-3), 141-145. doi:10.1016/s0304-3940(01)01636-6

Costa, R., Civello, D. A., Bernardinelli, E., Vanoni, S., Zopf, M., Scantamburlo, G., . . . Dossena, S. (2018). A Potassium-Selective Current Affected by Micromolar Concentrations of Anion Transport Inhibitors. Cell Physiol Biochem, 45(3), 867-882. doi:10.1159/000487282

Costa, R., Remigante, A., Civello, D. A., Bernardinelli, E., Szabo, Z., Morabito, R., . . . Dossena, S. (2020). O-GlcNAcylation Suppresses the Ion Current IClswell by Preventing the Binding of the Protein ICln to alpha-Integrin. Front Cell Dev Biol, 8, 607080. doi:10.3389/fcell.2020.607080

Dossena, S., Gandini, R., Tamma, G., Vezzoli, V., Nofziger, C., Tamplenizza, M., . . . Paulmichl, M. (2011). The molecular and functional interaction between ICln and HSPC038 proteins modulates the regulation of cell volume. J Biol Chem, 286(47), 40659-40670. doi:10.1074/jbc.M111.260430

Dossena, S., Maccagni, A., Vezzoli, V., Bazzini, C., Garavaglia, M. L., Meyer, G., . . . Paulmichl, M. (2005). The expression of wild-type pendrin (SLC26A4) in human embryonic kidney (HEK 293 Phoenix) cells leads to the activation of cationic currents. Eur J Endocrinol, 153(5), 693-699. doi:10.1530/eje.1.02018

Gandini, R., Dossena, S., Vezzoli, V., Tamplenizza, M., Salvioni, E., Ritter, M., . . . Furst, J. (2008). LSm4 associates with the plasma membrane and acts as a co-factor in cell volume regulation. Cell Physiol Biochem, 22(5-6), 579-590. doi:10.1159/000185542

Garavaglia, M. L., Bononi, E., Dossena, S., Mondini, A., Bazzini, C., Lanata, L., . . . Meyer, G. (2008). S-CMC-Lys protective effects on human respiratory cells during oxidative stress. Cell Physiol Biochem, 22(5-6), 455-464. doi:10.1159/000185494

Guizzardi, F., Rodighiero, S., Binelli, A., Saino, S., Bononi, E., Dossena, S., . . . Meyer, G. (2006). S-CMC-Lys-dependent stimulation of electrogenic glutathione secretion by human respiratory epithelium. J Mol Med (Berl), 84(1), 97-107. doi:10.1007/s00109-005-0720-y

Horn, R. (1991). Diffusion of nystatin in plasma membrane is inhibited by a glass-membrane seal. Biophys J, 60(2), 329-333. doi:10.1016/S0006-3495(91)82057-4

Kossler, S., Nofziger, C., Jakab, M., Dossena, S., & Paulmichl, M. (2012). Curcumin affects cell survival and cell volume regulation in human renal and intestinal cells. Toxicology, 292(2-3), 123-135. doi:10.1016/j.tox.2011.12.002

Li, Y., To, J., Verdia-Baguena, C., Dossena, S., Surya, W., Huang, M., . . . Torres, J. (2014). Inhibition of the human respiratory syncytial virus small hydrophobic protein and structural variations in a bicelle environment. J Virol, 88(20), 11899-11914. doi:10.1128/JVI.00839-14

Liu, W., Wei, Z., Ma, H., Cai, A., Liu, Y., Sun, J., . . . Seeram, N. P. (2017). Anti-glycation and anti-oxidative effects of a phenolic-enriched maple syrup extract and its protective effects on normal human colon cells. Food Funct, 8(2), 757-766. doi:10.1039/c6fo01360k

Mendanha, S. A., Anjos, J. L., Silva, A. H., & Alonso, A. (2012). Electron paramagnetic resonance study of lipid and protein membrane components of erythrocytes oxidized with hydrogen peroxide. Braz J Med Biol Res, 45(6), 473-481. doi:10.1590/s0100-879x2012007500050

Meyer, G., Rodighiero, S., Guizzardi, F., Bazzini, C., Botta, G., Bertocchi, C., . . . Paulmichl, M. (2004). Volume-regulated Cl- channels in human pleural mesothelioma cells. FEBS Lett, 559(1-3), 45-50. doi:10.1016/S0014-5793(04)00020-1

Morabito, R., Costa, R., Rizzo, V., Remigante, A., Nofziger, C., La Spada, G., . . . Dossena, S. (2017). Crude venom from nematocysts of Pelagia noctiluca (Cnidaria: Scyphozoa) elicits a sodium conductance in the plasma membrane of mammalian cells. Sci Rep, 7, 41065. doi:10.1038/srep41065

Tamma, G., Dossena, S., Nofziger, C., Valenti, G., Svelto, M., & Paulmichl, M. (2011). EGF stimulates IClswell by a redistribution of proteins involved in cell volume regulation. Cell Physiol Biochem, 28(6), 1191-1202. doi:10.1159/000335851
